# Excess urinary iodine concentration and thyroid dysfunction among school age children of eastern Nepal: a matter of concern

**DOI:** 10.1186/s13104-019-4332-y

**Published:** 2019-05-27

**Authors:** Man Kumar Tamang, Basanta Gelal, Binaya Tamang, Madhab Lamsal, David Brodie, Nirmal Baral

**Affiliations:** 1 Rotary Club of Dharan Ghopa, B. P. Koirala Institute of Health Sciences, Dharan, Nepal; 2 Department of Biochemistry, B. P. Koirala Institute of Health Sciences, Dharan, Nepal; 3 Bucks New University, Slough, UK

**Keywords:** Urinary iodine, Salt iodine, Deficiency, Children, Thyroid, Excess, Nepal

## Abstract

**Objectives:**

Deficiency as well as excess dietary iodine is associated with several thyroid disorders including Grave’s disease and goitre. Previously, cross sectional studies conducted among school children in Nepal showed high prevalence of iodine deficiency. In contrast, recently, few studies have revealed emerging trends of excess urinary iodine concentration in children. This paper, reports excess urinary iodine excretion and thyroid dysfunction among school age children from eastern Nepal.

**Results:**

It was a community based cross sectional study in which we measured urinary iodine excretion levels among school age children at baseline and after educational program. The educational program consisted of audio-visual and pamphlets on thyroid health. We also screened them for thyroid function status by physical examination and measuring serum thyroid hormones. Our results show that 34.4% of the children had excess urinary iodine concentration above the WHO recommended levels. Overall, 3.2% of the children were identified to have thyroid dysfunction. Urinary iodine concentration was significantly different between types of salt used and between salt iodine content categories.

## Introduction

Iodine deficiency disorders (IDD) are still major public health problems in developing countries although significant progress has been made towards their control strategies [1]. Urinary iodine is a sensitive marker of recent dietary iodine intake but not of thyroid dysfunction. Spot urine sample is generally recommended for population based studies [2–4]. Total goitre rate in Nepal has dramatically reduced to 0.4% in 2007 from 55% in 1965 [5]. Although iodine deficiency (ID) and prevalence of goitre have been well evidenced cases, association between excess iodine intake and thyroid disease have been found [6]. Few recently conducted studies in Nepal have demonstrated increasing trends of excess urinary iodine concentration (UIC) in children [6, 7]. In addition, latest Nepal National Micronutrient Status Survey 2016 has revealed that median urinary iodine concentration (mUIC) in 6–9 years children was above 300 µg/L [8]. This report along with few other studies point out possibility of chronic excess iodine intake in Nepalese children. Acute iodine excess in new-borns can cause hypothyroidism but limited data are available on the effects of chronic high intake of iodine in thyroid function [9]. The current study was aimed to assess iodine status among school age children (SAC) and correlate it with salt iodine content (SIC), type of salt used and gender of the children. Our other aim was to check improvement of urinary iodine excretion (UIE) levels after educational program.

## Main text

### Methods

This community based study approached 1042 SAC from twelve government schools. These schools were selected randomly from six Village Development Committees (VDC) of the Udaypur District, Province no. 1, Nepal such that two schools were included from each VDC. The study commenced in October 2015 and completed in July 2017. Spot urine samples and household salt samples were collected from 946 children. Blood samples were obtained from 245 children. The urine samples were used to determine UIC whereas salt samples were used to assess household SIC. The salt samples were classified as open type and packet (iodized) type. UIC was determined by the ammonium persulfate digestion microplate (APDM) method using the Sandell–Kolthoff reaction in a specially designed apparatus, sealing cassette (Hitachi, Japan) [10] in microplate format. SIC was determined by the iodometric titration method. Blood samples were analysed for thyroid hormones (free thyroxine and thyroid stimulating hormone). Thyroid stimulating hormone (TSH) and free thyroxine (fT4) were measured using the HUMAELISA kit (Human Diagnostics, Germany) following the manufacturer’s manual. Thyroid function status was determined according to the reference range provided in the kit, which reported TSH 0.39–6.16 mIU/L and fT4 0.8–2.2 ng/dL. Sub clinical hypothyroidism is considered if TSH > 6.16 mIU/L with fT4 in the normal range whereas subclinical hyperthyroidism is considered if TSH < 0.39 mIU/L with fT4 in the normal range. Ethical approval for this research was taken from Institutional Review Committee, B. P. Koirala Institute of Health Sciences (BPKIHS) (IRC/655/015). Informed consent was taken from school teacher, head teacher and concerned guardians before samples were collected. To compare mean difference between two groups, Mann Whitney U test was used while for comparing three groups, Kruskal Wallis test was used.

### Educational program

After the baseline survey, a series of educational programs were organized targeting the schools. The educational program consisted of an awareness of iodine and thyroid through pamphlets, posters and audio-visual aids. Information on iodized salts use and its importance in health was provided to the SAC. The school teachers were trained on iodine, thyroid diseases, its preliminary identification in community setting and the importance of iodized salt. These programmes were organized twice in each school. After 1 year of the educational program, we again assessed UIC in the same cohort of school children, achieving a total of 311 urine samples.

### Results

**Fig. 1:**
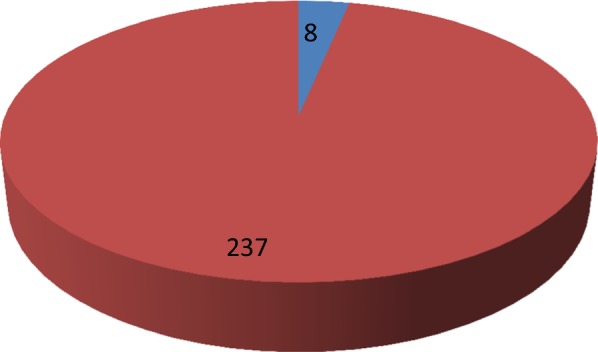
Thyroid function status among the school children (n = 245). Blue colour shows number of school children suffering from any form of thyroid dysfunction (either hypothyroidism or hyperthyroidism). Red colour shows number of children with normal thyroid function status

Results showed that mUIC among the children at baseline was 231 µg/L (IQR: 156–333) which lies in the more than adequate level. We found that 9.8% of the SAC had iodine deficiency; followed by 23.5% of the SAC who had more than adequate UIC. Most importantly, 34.4% of children had excess UIC (i.e. ≥ 300 µg/L). Table 1 provides the full details. Open type salt was used by 9.3% of the children while majority of them used iodized salt distributed by Salt Trading Corporation Nepal. Salt iodine content (SIC) determination showed that 11.8% of the salt samples had iodine content of 0–15 ppm whereas 88.2% contained more than 15 PPM. As presented in Table 2, UIC was not significantly different between boys and girls (p = 0.64), but significantly different between the SIC categories (p < 0.05) and between type of salt used (p < 0.05). As shown in Fig. 1, 3.2% of the SAC had thyroid dysfunction with either hypothyroidism or hyperthyroidism. The assessment of thyroid hormones revealed that 1.6% of the SAC had subclinical hyperthyroidism and one child had overt hyperthyroidism. Overt and subclinical hypothyroidisms were identified in two children and one child respectively. Further, urinary iodine measurement after the educational program revealed that the proportion of excess UIC slightly decreased to 32.8% from baseline of 34.4%. After one year of educational program, mUIC in school children (n = 311) was 201 µg/L, having found to decrease from baseline of 231 µg/L.

**Table 1:** UIC among the school children (n = 946, before and n = 311 after educational program

Urinary iodine concentration (UIC) status	Before educational program		After educational program	
	Frequency (n)	Percentage	Frequency (n)	Percentage
Severe ID ( < 20 µg/L)	4	0.4	2	0.6
Mild ID (20–49 µg/L)	22	2.3	12	3.9
Moderate ID (50–99 µg/L)	67	7.1	47	15.1
Adequate (100–199 µg/L)	306	32.3	94	30.2
More than adequate (200–299 µg/L)	222	23.5	54	17.4
Excess ( ≥ 300 µg/L)	325	34.4	102	32.8
Total	946	100	311	100

**Table 2:** Comparison of UIC within SIC, gender and type of salt used

Variables	Categories	Mean rank	p-value
UIC	Salt iodine content (SIC) (ppm)		0.000^a^
	0	191.02	
	< 15	241.54	
	> 15	485.43	
UIC	Gender		0.640^b^
	Boy	469.16	
	Girl	477.47	
UIC	Type of salt used		0.000^b^
	Open salt	169.79	
	Packet salt	483.52	

^a^Kruskal Wallis test

^b^Mann–whitney U test

### Discussion

The present study highlighted excess urinary iodine excretion levels in SAC from eastern Nepal. Sufficient sample size is one of the big strengths of our study. In addition, physical examination of the children and biochemical test further strengthen our results. Similar to our findings, two studies from Nepal have reported high prevalence of excess UIC among school children with one study reporting median UIC of 291.8 µg/L (IQR 181.3–411.5 µg/L [7, 9]. A survey conducted in Nepal in 2007 demonstrated mUIC of 203 µg/L [11]. A cross sectional study from India in 2013 found a similar mUIC of 200 µg/L [12]. This implies that the population is in transition phase from iodine deficiency to iodine sufficiency. The latest Iodine Global Network report has highlighted several countries around the world to be categorized as having excess iodine status in population [11].

The time has now come to monitor the iodine status at national level and adjust policies accordingly. Together with our current results, consistent findings of excess UIC among school children imply chronic exposure to high dietary iodine intake. A reason for this may be due to poor awareness among SAC on the optimal consumption of iodized salt. Secondly, awareness regarding consumption of iodized salt among the households need to be improved with regard to optimal intake. In addition, poor monitoring of salt distribution and household salt consumption after initiation of salt iodization program by the government is probably a significant factor underlying this outcome. This is a matter of concern as high intake of dietary iodine could result in several thyroid disorders. This warrants a further large scale cross sectional as well as cohort studies comprising several districts to assess the iodine status of the population of the country.

### Conclusion

Based on our results, we conclude that there is a high prevalence of excess urinary iodine concentration among school age children of eastern Nepal. Since almost 88% of the household salt samples tested were found to have SIC of more than 15 PPM, we suggest the young children and their family to consume optimal salt in their diet. This may prevent them from iodine induced thyroid disorders. Also, the study recommends to explore further regarding excess iodine status in nationally representative population. Our findings suggest that programs and policies should be developed to explore and address this issue further.

## Limitations

The study has a number of limitations. First, we could not collect blood samples from all the participating school children due to refusal from their parents. Second, reassessment of urinary iodine concentration after educational program could only enrol about 30% baseline population, which might limit its generalization. Third, free tri-iodothyronine (fT3) concentration was not measured in the blood samples.

## Data Availability

The data set used and analysed in this study are available from the corresponding author upon reasonable request.

## Abbreviations

UIC: urinary iodine concentration
SIC: salt iodine content
UIE: urinary iodine excretion
SAC: school age children
IDD: iodine deficiency disorder
VDC: village development committee
APDM: ammonium persulfate digestion microplate
BPKIHS: B. P. Koirala Institute of Health Sciences

## References

[1] Zimmermann M, Jooste P, Pandav C (2008). Iodine-deficiency disorders. Lancet.

[2] Delange F, Burgi H, Chen Z, Dunn J (2002). World status of monitoring iodine deficiency disorders control programs. Thyroid.

[3] Assessment of iodine deficiency disorders and monitoring their elimination: a guide for programme managers, 3rd edition. Geneva: World Health Organization, International Council for Control of Iodine Deficiency Disorders and UNICEF. https://www.who.int/nutrition/publications/micronutrients/iodine_deficiency/9789241595827/en/. Accessed 5 Dec 2018.

[4] Zimmermann M, Anderson M (2012). Assessment of iodine nutrition in populations: past, present, and future. Nutr Rev.

[5] Gelal B, Baral N. Moving toward the sustainable elimination of IDD in Nepal. IDD NEWSLETTER; 2010. https://www.ign.org/idd-newsletter-22010.htm. Accessed 25 Dec 2018.

[6] Leung A, Braverman L (2014). Consequences of excess iodine. Nat Rev Endocrinol.

[7] Shakya P, Gelal B, Das B, Lamsal M, Pokharel P, Nepal A, Brodie D, Sah G, Baral N (2015). Urinary iodine excretion and thyroid function status in school age children of hilly and plain regions of Eastern Nepal. BMC Res Notes.

[8] Ministry of Health and Population, Nepal; New ERA; UNICEF; EU; USAID; and CDC. National Nepal Micronutrient Status Survey 2016. Kathmandu, Nepal. 2018. https://www.unicef.org/nepal/reports/nepal-national-micronutrient-status-survey-report-2016. Accessed 15 Dec 2018.

[9] Nepal A, Suwal R, Gautam S, Shah G, Baral N, Andersson M, Zimmermann M (2015). Subclinical hypothyroidism and elevated thyroglobulin in infants with chronic excess iodine intake. Thyroid.

[10] Ohashi T, Yamaki M, Pandav C, Karmarkar M, Irie M (2000). Simple microplate method for determination of urinary iodine. Clin Chem..

[11] Iodine Global Network. Global Score card of Iodine Nutrition in 2017 in the general population and in pregnant women (PW). IGN, Zurich, Switzerland. 2017. https://www.ign.org/scorecard.htm. Accessed 16 Dec 2018.

[12] Kapil U, Pandey R, Kabra M, Jain V, Sareen N, Bhadoria A, Vijay J, Nigam S, Khenduja P (2013). Status of iodine deficiency in district Kangra, Himachal Pradesh after 60 years of salt iodization. Eur J Clin Nutr.

